# Metabolomics-Based Profiling of *Clerodendrum speciosum* (Lamiaceae) Leaves Using LC/ESI/MS-MS and In Vivo Evaluation of Its Antioxidant Activity Using *Caenorhabditis elegans* Model

**DOI:** 10.3390/antiox11020330

**Published:** 2022-02-08

**Authors:** Fadia S. Youssef, Mansour Sobeh, Malak Dmirieh, Hanin A. Bogari, Abdulrahman E. Koshak, Michael Wink, Mohamed L. Ashour, Sameh S. Elhady

**Affiliations:** 1 Department of Pharmacognosy, Faculty of Pharmacy, Ain-Shams University, Abbasia, Cairo 11566, Egypt; 2 AgroBioSciences, Mohammed VI Polytechnic University, Lot 660–Hay MoulayRachid, Ben-Guerir 43150, Morocco; mansour.sobeh@um6p.ma (M.S.); wink@uni-heidelberg.de (M.W.); 3 Institute of Pharmacy and Molecular Biotechnology, Heidelberg University, Im Neuenheimer Feld 364, 69120 Heidelberg, Germany; dmirieh@stud.uni-heidelberg.de; 4 Department of Pharmacy Practice, Faculty of Pharmacy, King Abdulaziz University, Jeddah 21589, Saudi Arabia; hbogari@kau.edu.sa; 5 Department of Natural Products, Faculty of Pharmacy, King Abdulaziz University, Jeddah 21589, Saudi Arabia; aekoshak@kau.edu.sa (A.E.K.); ssahmed@kau.edu.sa (S.S.E.); 6 Department of Pharmaceutical Sciences, Pharmacy Program, Batterjee Medical College, Jeddah 21442, Saudi Arabia

**Keywords:** anti-inflammatory, antioxidant, *Caenorhabditis elegans*, *Clerodendrum speciosum*, molecular modelling, phytoconstituents, Lamiaceae

## Abstract

We investigated the antioxidant activity of the total methanol extract of *C. speciosum* leaves (CST), the ethyl acetate (CSE), and the remaining aqueous (CSR) fractions in vitro, in vivo using *Caenorhabditis elegans* model, and in silico. LC-ESI-MS/MS analysis was employed for metabolic profiling of CST. ADME/TOPAKT prediction was performed to determine the potential pharmacokinetic, pharmacodynamic, and toxicity properties of the major identified phytoconstituents. All examined samples showed considerable antioxidant activity where CST, CSE, and CSR displayed EC_50_ values of 27.1, 16.2, and 21.3 µg/mL, respectively, in 2,2-diphenyl-1-picrylhydrazyl (DPPH^•^) assay, whereas they showed 11.44, 16.27, and 12.16 Fe^2+^ equivalents/mg of sample, respectively, in ferric reducing antioxidant power (FRAP) assay. CST, CSE, and CSR displayed total phenolic content of 262, 326, and 289 mg GAE/g sample, respectively. In vivo antioxidant study revealed that CST at 150 μg/mL increased the survival rate of *C. elegans* by 71.88% compared to untreated group. Regarding intracellular reactive oxygen species (ROS), worms treated with 150 μg/mL of CSE exhibited 60.42% reduction of ROS compared to the untreated group. Quantitation of hsp-16.2/GFP expression in *Caenorhabditis elegans* showed that worms treated with 150 μg/mL of CSR exerted 40.43% reduction in fluorescence with respect to the untreated group. LC-ESI-MS/MS of CST revealed the presence of sixteen secondary metabolites belonging mainly to polyphenolics with phenyl propanoids constituting the major detected class. The in silico study showed that rosmarinic acid displayed the best fitting within the active sites of Daf-2 protein with considerable safety profile and limited pharmacokinetic and pharmacodynamic that could be slightly enhanced by certain treatment.

## 1. Introduction

Oxidative stress occurs due to a marked imbalance between the amount of free radicals and natural antioxidants inside the human body that ultimately leads to cell and tissue damage. During metabolic processes, the body normally produces free radicals that are accompanied by the generation of naturally occurring antioxidants that counteract and neutralize the excessive production of free radicals. In contrast, environmental factors such as pollution and radiation in addition to an unhealthy lifestyle greatly increase oxidative stress [1,2]. The uncontrolled generation of free radicals vigorously triggers inflammation with a concomitant production of additional free radicals that further aggravates the oxidative stress. ROS can also cause mutations to DNA which can lead to several gene-based diseases such as cardiovascular disease, diabetes mellitus, neurodegenerative disorders, allergy, as well as cancer [3,4]. 

*Caenorhabditis elegans* is a nematode that has been introduced in the 1970s by Sydney Brenner in the biological research serving as a model organism. Since then its utilization to evaluate the efficacy of many drugs to counteract oxidative stress, inflammation, ageing, and neurodegeneration has been widely adopted [5,6]. This is mainly attributed to the presence of a great degree of similarity between *C. elegans* and various human pathways, and thus the worm provides acceptable probabilities to explore mode of action and medicinal values of phytoconstituents obtained from plants as well as foods [7,8,9]. 

*Clerodendrum* (formerly Verbenaceae, now Lamiaceae) comprises about 150 species mostly of shrubs, small trees, as well as herbs. They are native to the tropics in addition to the warm temperate areas around the world. Several species are popular garden plants. The different organs of its members are highly popular for the curing of various ailments including inflammatory disorders, asthma, rheumatism, skin diseases, cough, malaria, in addition to possessing febrifuge and vermifuge effects. The genus produces different secondary metabolites, mainly flavonoids, sterols, phenolics, and essential oils that are undoubtedly responsible for the observed biological properties [10]. 

*C. speciosum* represents a hybrid between *C. splendens*, the Scarlet glory-bower, and *C. thomsoniae*, the Bleeding heart glory-bower having red violet flowers with gamosepalous calyx resembling *C. thomsoniae.* The former was documented to have analgesic, anti-inflammatory, antispasmodic cardio-protective, immune modulatory, oxytocic, and sedative properties [11]; however, the latter was reported to alleviate headaches convulsions, epilepsy, as well as parasitic diseases with various phytoconstituents isolated from both species [12].

Although many biological activities and phytochemicals have been investigated for both *C. thomsoniae* and *C. splendens,* phytochemical data are missing for *C. speciosum.* Hence, in the forgoing study, the antioxidant activity of the total methanol extract of *C. speciosum* leaves as well as ethyl acetate and remaining aqueous fractions were assessed using in vitro antioxidant assays as 2,2-diphenyl-1-picrylhydrazyl (DPPH) and ferric reducing antioxidant power (FRAP) in addition to the determination of the Total Phenolic Content (TPC). Besides, the ability to combat oxidative stress was also assessed in vivo using *Caenorhabditis elegans* model for the first time. Moreover, the predominant phytochemicals in the total methanol extract of *C. speciosum* leaves were tentatively characterized using LC-ESI-MS/MS technique that probably explains the reason underlying the observed activity. In addition, a theoretical ADME/TOPAKT prediction was performed to the predominant identified phytoconstituents aiming to determine their potential pharmacokinetic, pharmacodynamic, and toxicity properties. 

## 2. Materials and Methods

### 2.1. Plant Material

*Clerodendrum speciosum* (Lamiaceae) leaves were collected from Mazhar Botanical Garden that is located 30 km on the Cairo-Alexandria road in Egypt in April 2018. The plant was kindly authenticated and identified via inspection of the morphological features by Eng. Terase Labib, Consultant of Plant Taxonomy at the Ministry of Agriculture and El-Orman Botanical Garden, Giza, Egypt. Voucher specimen from the collected plant was maintained at the Pharmacognosy Department, Faculty of Pharmacy, Ain Shams University, giving the code PHG-P-CS 156.

### 2.2. Preparation of the Extract and Different Fractions of C. speciosum Leaves

A total of 2 kg of *C. speciosum* leaves were air-dried and crushed into coarse powder to give 200 g. They were macerated in distilled methanol (3 L × 3) followed by their filtration. Consequently, the filtrate was completely evaporated under reduced pressure at 45 °C using Rotary evaporator (Heidolph, Germany) until complete dryness and is followed by lyophilization to give 19.43 g of dried total methanol extract (CST). The lyophilized powder was re-dissolved in 70% methanol for fractionation using 3 L of *n*-hexane, 1 L of dichloromethane, and 1 L of ethyl acetate successively. Drying of the obtained fractions was performed under reduced pressure at 45 °C to give 4.2 g of *n*-hexane, 1.5 g of dichloromethane, and 4.75 g of ethyl acetate (CSE) dried fractions meanwhile the remaining hydro methanol fraction (CSR) weighed about 7.48 g.

### 2.3. Determination of Total Phenolic Content of the Extract and Different Fractions of C. speciosum Leaves

Total phenolic content was determined for CST, CSE and CSR using Folin–Ciocalteu method that was previously reported by Zhang et al. [13]. Briefly, 20 µL of each of the tested samples (5 mg/mL) were used in this assay and are used to fill a 96-well microplate followed by the addition of 100 µL of Folin–Ciocalteu reagent. Then, the tested samples were maintained at room temperature for 5 min, subsequently followed by the addition of 80 µL of a 7.5% sodium carbonate solution and mixed well. Finally, the plate was maintained for 2 h at room temperature in the dark followed by the determination of absorbance using Biochrom Asys UVM 340 Microplate Reader at 750 nm. Meanwhile, gallic acid was serially diluted in concentration range (0–200 µg/mL) to be used as a standard for the construction of the calibration curve. The total phenolic content of the examined samples was expressed in terms of gallic acid equivalent (GAE)/g sample.

### 2.4. In Vitro Antioxidant Determination of the Extract and Different Fractions of C. speciosum Leaves

#### 2.4.1. 2,2-Diphenyl-1-picrylhydrazyl Radicle (DPPH^•^) Scavenging Capacity Assay

DPPH^•^ (2,2-diphenyl-1-picrylhydrazyl) radical scavenging activity of the tested samples was evaluated employing the standard method previously reported by Blois [14] with certain modifications using a 96-well microplate. Briefly, 100 μL of DPPH solution (200 µM) were added to 100 µL within the concentration range (1.25–50 μg/mL). Incubation of the samples was performed at room temperature for 30 min and consequently the absorbance was measured at 517 nm by Tecan Safire II ^TM^ spectrophotometer. The % of DPPH scavenging capacity was calculated using the following equation:

DPPH scavenging effect (%) = [(A0 − A1)/A0] × 100

A0 and A1 represent the absorbance of the negative control and the tested sample, respectively. The measurements were performed in triplicates where EC_50_ value was determined by sigmoid non-linear regression employing the GraphPad Prism version 5 software (GraphPad Software, Inc., La Jolla, CA, USA).

#### 2.4.2. Ferric Reducing Antioxidant Power (FRAP) Assay

Ferric reducing antioxidant assay was done as previously reported by Benzie et al. [15] with certain modification to a 96-well microplate. The assay relied on the tendency of the tested samples to exert reduction of the ferric complex (2,4,6-tripyridyl-s-triazine–Fe^3+^-TPTZ) to ferrous form (Fe^2+^-TPTZ) when experiencing low pH. Briefly, the FRAP working solution is prepared by mixing of 300 mM acetate buffer (pH 3.6), 20 mM FeCl_3_.6 H_2_O, and 10 mM TPTZ (2,4,6-tripyridyl-s-triazine) in 40 mM HCl with a ratio of 10:1:1 before analysis. Warming of the fresh FRAP working solution was performed for 30 min at 37 °C before the assay. Meanwhile, FeSO_4_.7H_2_O was used as a standard. Then, an addition of 175 µL of freshly prepared FRAP working solution to 25 µL of the prepared samples was performed at 37 °C and maintained for 7 min. Absorbance was measured at 595 nm by Tecan Safire II ^TM^ spectrophotometer where reduction is evidenced by the appearance of intense blue color. All the measurements were performed in triplicates and results were expressed as Fe^2+^ equivalents/mg of sample. 

### 2.5. In Vivo Antioxidant Determination of the Extract and Different Fractions of C. speciosum Leaves Using C. elegans

#### 2.5.1. *C. elegans* Strains and Culture Conditions

Two types of nematodes, namely, the transgenic strain TJ375 (hsp-16.2/GFP) and the wild type N2 strains, were obtained from the *Caenorhabditis* Genetic Center (CGC) and were kept under standard conditions. They were maintained at 20 °C on nematode growth medium (NMG) inoculated with living *Escherichia coli* (OP50). All utilized worms were age-synchronized and this was achieved via the treatment of gravid adults with sodium hypochlorite where the eggs were permitted to hatch in M9 buffer and subsequently the obtained larvae was transmitted to S-medium supplemented with living *Escherichia coli* (OP50) [16].

#### 2.5.2. Survival Assay of *C. elegans* Using Juglone Induced Oxidative Stress

Juglone at a concentration (80 µM) was used to induce oxidative stress in synchronized worms which are N2 strain as well as L1 larvae stage that were brought up in S-media supplemented with living *E. coli* OP50 as a food source at 20 °C. These worms were subsequently treated with 50, 100, and 150 μg/mL of CGT, CSE, and CSR for 48 h except the control group that received no treatment. Meanwhile, epigallocatechin gallate (EGCG) at a concentration of 100 μg/mL was used as a positive control. Consequently, addition of 80 µM of juglone was performed to elicit oxidative stress followed by counting the survivors after 24 h. The survival rate was calculated as percentage of the live worms using one-way ANOVA and subsequently followed by Bonferroni (post-hoc) correction [17].

#### 2.5.3. Assessment of Reactive Oxygen Species (ROS) in *C. elegans*

Synchronized worms, N2 strain, and L1 larvae stage, that were brought up in S-media supplemented with living *E. coli* OP50 as a food source at 20 °C, were subsequently treated with 50, 100 and 150 μg/mL of CST, CSE, and CSR for 48 h except for the control group that received no treatment. Careful washing of the worms was achieved by M9 buffer followed by their transfer and incubation in 1 mL of CM-H2DCF-DA (20 µM) at 20 °C for 30 min. Removal of excess dye was achieved via washing the worms again by M9 buffer followed by their analysis with fluorescence microscopy (λ _Excitation_ 480/20 nm; λ _Emission_ 510/38 nm). Paralysis of the worms was accomplished by the addition of 10 mM of sodium azide which were then placed on a glass slide. Images were captured at fixed exposure time from at least 30 worms. The whole body relative fluorescence was estimated using the Image J software densitometrically and the results were expressed as the mean pixel intensity (mean ± SEM) and compared using one-way ANOVA and subsequently followed by Bonferroni post-hoc correction [18].

#### 2.5.4. Quantitation of hsp-16.2/GFP Expression in *Caenorhabditis elegans*

Synchronized worms of the transgenic strain TJ375 that express hsp-16.2/GFP) were brought up in S-media supplemented with living *E. coli* OP50 as a food source at 20 °C. They were subsequently treated with 50, 100, and 150 μg/mL of CST, CSE, and CSR for 48 h except the control group that received no treatment. Exposure of these worms to oxidative stress was achieved by adding 20 µM of juglone for 24 h and consequently the worms were analyzed using fluorescence microscopy (λ _Excitation_ 480/20 nm; λ _Emission_ 510/38 nm). Hsp-12.6 promoter that linked to gene encoding GFP (green fluorescence protein) exists in the mutant strain, TJ375, and its expression is directly determined quantitatively via determining fluorescence intensity of the GFP reporter that exists in the pharynx of the worm. Then the worms were treated with 10 mM of sodium azide to reduce the motility of the worms which were then placed on a glass slide. Images were captured by 20X objective lens at fixed exposure time from at least 30 worms. The pharynx relative fluorescence was estimated using the Image J software densitometrically and the results were expressed as the mean pixel intensity (mean ± SEM) and compared using one-way ANOVA and subsequently followed by Bonferroni (post-hoc) correction.

### 2.6. Phytochemical Profiling of C. speciosum Leaves Total Methanol Extract Using LC/ESI/MS-MS

An analysis of a total methanol extract from *C. speciosum* leaves was carried out using HPLC Agilent 1200 series instrument. The used column was Gemini C18 with 3 µm and 110 Å obtained from Phenomenex possessing 100 × 1 mm i.d as dimensions. This is in turn is protected by a guard column formed of RP C18 100 Åpossessing 5 mm × 300 µm i.d., 5 µm as dimensions. Elution was done in a gradient manner at a flow rate of 50 µL/min where the mobile phase is composed of 2% acetic acid (A) and 90% MeOH in 2% acetic acid (B). The elution started with 5% B at 0 min that gradually increased to 50% B in 60 min and then consequently elevated to 90% B within 60 min and maintained for 5 min. Mass spectrometry was performed via the utilization of Fourier transform ion cyclotron resonance mass analyzer supplemented with anelectrospray ionization (ESI) system. Controlling of the system was done using the X-calibur^®^ software where the data were gathered in the negative ion mode as previously described by Sobeh et al. The mass in the range of 150 to 2000 *m/z* was selected for the full mass scan with resolution up to 100,000 [19].

### 2.7. Computer Aided Drug Design Studies

#### 2.7.1. In Silico Molecular Docking

To get a deep insight into the molecular antioxidant effect of the identified compounds from *C. speciosum* leaves total methanol extract in *Caenorhabditis elegans,* molecular docking was done for all of them against daf-2 protein (PDB ID: 4JDE; 1.90Å) obtained from *Caenorhabditis elegans* using Discovery Studio 4.5 (Accelrys Inc., San Diego, CA, USA) employing C-Docker protocol. The X-ray crystal structure of the protein used in this study was obtained from protein data bank in pdb format. The default protocol for the preparation of protein implemented in Discovery Studio 4.5 (Accelrys Inc., San Diego, CA, USA) was utilized for the preparation of the studied protein. This was achieved by removal of water molecules, addition of hydrogen atoms followed by cleaning of the protein from any unwanted interactions. CHARMm was selected as the forcefield; however, MMFF94 was chosen for calculation of partial charge and then minimization of the added hydrogen in nearly 2000 steps. The active centers were selected relied upon the previous data approaching the catalytic domain. ChemDraw 13.0 was used to construct the 2D structures of the compounds and then they were converted to 3D using default ligand preparation protocol constructed Discovery Studio 4.5 (Accelrys Inc., San Diego, CA, USA). Then, docking of the prepared compounds in the active center was done employing C-Docker protocol. CHARMm force field was determined and the binding energies were computed using distance dependent dielectric implicit solvation model for the selected docking poses. Meanwhile the following equation was used to determine binding energies (∆G) [3,20].

Δ*G*_binding_ = E_complex_ − (E_protein_ + E _ligand_),

where Δ*G*_binding_: The ligand–protein interaction binding energy; E_complex_: The potential energy for the complex of protein bound with the ligand; E_protein:_ The potential energy of protein alone; and E_ligand_: The potential energy for the ligand alone.

#### 2.7.2. ADME/TOPAKT Prediction

The identified compounds from *C. speciosum* leaves total methanol extract were subjected to AMET prediction (absorption, distribution, metabolism, excretion, and toxicity) in addition to toxicity determination (TOPKAT) using Discovery Studio 4.5 (Accelrys Inc., San Diego, CA, USA). This was performed in an effort to evaluate their pharmacokinetic, pharmacodynamic and toxicity behavior. Blood–brain barrier penetration (BBB), human intestinal absorption (HIA), aqueous solubility, plasma protein binding prediction (PPB), hepatotoxicity level, and cytochrome P450 2D6 were selected as ADMET descriptors. Meanwhile, the carcinogenic effect on male and female rat NPT (National Toxicology Program), Ames mutagenicity, eye and dermal irritation, and Chronic LOAEL were chosen as TOPKAT parameters [21,22].

## 3. Results and Discussion

### 3.1. Determination of the Total Phenolic Content of C. speciosum Leaves Extract and Different Fractions

The total phenolic content (TPC) of *C. speciosum* leaves extract and different fractions, namely, CST, CSE, and CSR was determined using Folin–Ciocalteu method. All of the tested samples showed considerable values of total phenolic content with the ethyl acetate fraction (CSE) showed the highest value followed by the remaining hydro methanol fraction (CSR) and CST where they showed TPC values of 326 ± 9.7, 289 ± 6.8, and 262 ± 3.8 mg GAE/g sample, respectively. Phenolic compounds possess redox features that enable them to act as antioxidants. The presence of free hydroxyl groups in the phenolic compounds enhanced their free radical scavenging potential and thus the total phenolic content determination could serve as a primary marker that overview the antioxidant capacity of the tested sample [23,24,25].

### 3.2. In Vitro Antioxidant Determination of the Extract and Different Fractions of C. speciosum Leaves

Exposure to stress, chemical substances as well as smoking markedly exaggerates oxidative stress causing the generation of a huge amount of free radicals. This in turn triggers the occurrence of many life threatening diseases exemplified by cancer, diabetes, neurological, and cardiovascular disorders in addition to exacerbating ageing [17]. Thus, in vitro antioxidant evaluation of CST, CSE, and CSR was performed using 2,2-diphenyl-1-picrylhydrazyl radicle (DPPH^•^) scavenging capacity assay as well as ferric reducing antioxidant power (FRAP) assay. These assays are highly used to assess the antioxidant potential of plant extracts, their pure isolated compounds in addition to food products as their long-lived radicals represented by DPPH^•^ and FeSO_4_ are reliable and sensitive [26]. Concerning 2,2-diphenyl-1-picrylhydrazyl radicle (DPPH^•^) scavenging capacity assay, it relied upon bleaching the color of DPPH methanol solution (purple) where it converted gradually to yellow color depending on the antioxidant potential of the tested sample. Thus, the results are highly correlated to the antioxidant capacity of the examined sample where antioxidants possess the ability to donate hydrogen atom or electron that consequently reduces the radical solutions [27]. Besides, FRAP is an easy, rapid, and reproducible method that accurately assesses the reduction of ferric ion (Fe^3+^) to ferrous (Fe^2+^) that is perfectly linked to the antioxidant power of phytoconstituents and is determined spectrophotometrically [22]. Results displayed in Table 1 revealed that all of the tested samples showed marked antioxidant ability in both assays with the ethyl acetate fraction (CSE) exhibited the highest activity. CST, CSE, and CSR displayed EC_50_ values of 27.1, 16.2, and 21.3 µg/mL, respectively, in DPPH^•^ assay, whereas they showed antioxidant capacity estimated by 11.44, 16.27, and 12.16 Fe2^+^ equivalents/mg of sample, respectively. 

### 3.3. In Vivo Antioxidant Determination of the Extract and Different Fractions of C. speciosum Leaves Using Caenorhabditis elegans Model

#### 3.3.1. Survival Assay of *Caenorhabditis elegans* Using Juglone Induced Oxidative Stress

**Figure 1:**
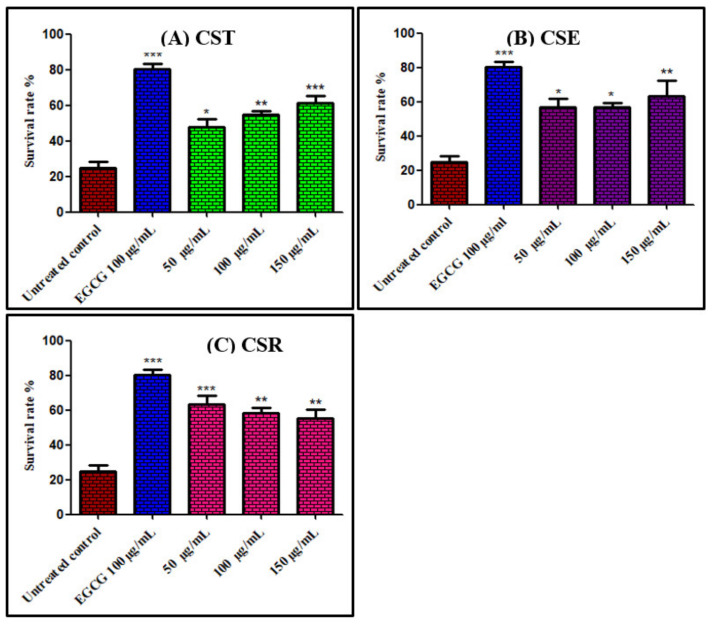
Survival rate of *Caenorhabditis elegans* pre-treated with different concentrations (50, 100, and 150 μg/mL) of extracts and fractions of *C. speciosum* leaves; (**A**) CST, (**B**) CSE, and (**C**) CSR; untreated control receives juglone treatment; EGCG = Epigallocatechin gallate (100 μg/mL) after juglone treatment; CST, CSE, and CSR groups receive (50, 100, and 150 μg/mL) of CST, CSE, and CSR after juglone treatment; * *p* < 0.05, ** *p* < 0.01, and *** *p* < 0.001.

Juglone, 5-hydroxy-1,4-naphthoquinone, is isolated from *Juglans regia* and is considered as a natural toxin that elicits oxidative stress acting as pro-oxidant. Thus, exposure to a high concentration of juglone is drastic to *Caenorhabditis elegans* that promptly causes its death meanwhile the presence of antioxidant agents that capture free radicals possibly hinder this effect [5,28]. Results displayed in Figure 1 showed that *C. elegans* pretreated with different concentrations (50, 100, and 150 μg/mL) of extracts and fractions of *C. speciosum* leaves markedly improved the survival rate of the nematode. *C. speciosum* total methanol extract (CST) at 150 μg/mL exerted the highest in vivo antioxidant potential as evidenced by the high survival rate estimated by 71.88% compared to untreated control group that received juglone alone (24.82% survival rate) and approaching that pretreated with 100 μg/mL of EGCG (epicogallocatechin gallate) that exerted 80.26% survival rate. From Figure 1 it is clearly obvious that CST, CSE and CSR exerted 64.44%, 56.59%, and 63.43% survival rate at 50 µg/mL, respectively; meanwhile, they showed 65.03%, 56.78%, and 57.72% survival rate at 100 µg/mL, respectively, whereas they exhibited 71.88%, 63.53%, and 55.85% survival rate at 150 µg/mL, respectively. It is worthy to highlight that the decreased in the survival rate in the group pretreated with CSR by increasing the concentration of the administered drug reflects its increased toxicity at high concentrations, besides all the tested samples revealed toxicity at 200 µg/mL. 

#### 3.3.2. Assessment of Intracellular Reactive Oxygen Species (ROS) in *Caenorhabditis elegans*

**Figure 2:**
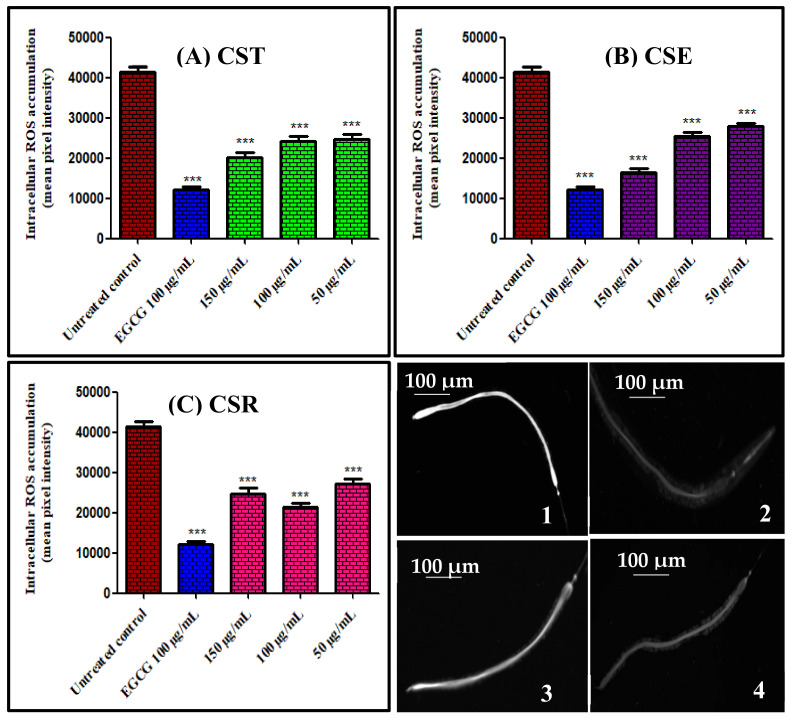
Intercellular ROS accumulation in *Caenorhabditis elegans* pre-treated with different concentrations (50, 100, and 150 μg/mL) of extracts and fractions of *C. speciosum* leaves; (**A**) CST, (**B**) CSE, and (**C**) CSR estimated as mean pixel intensity. Images of *C. elegans* by fluorescence microscopy (1) untreated control group, (2) pre-treated with 100 μg/mL of EGCG, (3) pre-treated with 50 μg/mL of CSR, and (4) pre-treated with 150 μg/mL of CSE; untreated control receives juglone treatment; EGCG = Epigallocatechin gallate (100 μg/mL) after juglone treatment; CST, CSE, and CSR groups receives (50, 100, and 150 μg/mL) of CST, CSE, and CSR after juglone treatment; *** *p* < 0.001.

In order to evaluate the amount of reactive oxygen species (ROS) within the cells that in turn help in the determination of the in vivo antioxidant potential of the administered samples, 2′,7′-dichlorofluorescindiacetate (CMH_2_DCF-DA), which acts as a membrane permeable reagent, was utilized. The presence of intracellular esterases causes the deacetylation of the reagent to a non-fluorescent compound that consequently oxidized in the presence of ROS, in particular H_2_O_2_ producing 2′,7′-dichlorofluorescein (DCF), a compound with high fluorescence that can be analyzed using fluorescence microscopy. Herein treatment of the worms with different concentrations (50, 100, and 150 μg/mL) of extract and fractions of *C. speciosum* leaves for 48 h resulted in a notable reduction in the concentration of intracellular ROS as evidenced by fluorescence microscopy analyses in a dose dependent manner in most of the samples that in turn reflects their ability to scavenge ROS in vivo (Figure 2). Images revealed that worms treated with 150 μg/mL of CSE experienced the lower fluorescence intensity when compared to untreated control group showing 60.42% reduction in fluorescence compared to untreated control group and approaching that pretreated with 100 μg/mL of EGCG (epigallocatechin gallate) that revealed 70.44% decrease in fluorescence. CST, CSE, and CSR exerted 41.21%, 36.59%, and 34.34% reduction in fluorescence compared to untreated group 50 μg/mL, respectively; meanwhile, they showed 41.49%, 38.79%, and 48.16% reduction in fluorescence compared to untreated group 100 μg/mL, respectively, whereas they exhibited 51.67%, 60.42%, and 40.38% reduction in fluorescence compared to untreated group 150 μg/mL, respectively. From the displayed results it is obvious that CSR at 50 μg/mL displayed the lowest reduction in fluorescence with respect to all of the examined samples meanwhile it showed higher reduction in fluorescence at 100 μg/mL than 150 μg/mL that may indicate its toxicity at a high concentration.

#### 3.3.3. Quantitation of hsp-16.2/GFP Expression in *Caenorhabditis elegans*

**Figure 3:**
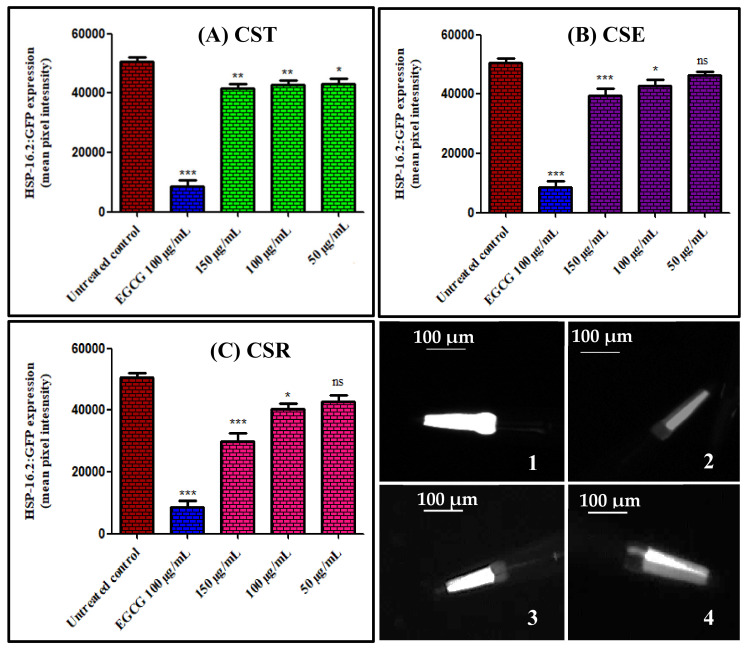
Hsp-16.2/GFP expression in *Caenorhabditis elegans* pre-treated with different concentrations (50, 100, and 150 μg/mL) of extracts and fractions of *C. speciosum* leaves; (**A**) CST, (**B**) CSE, and (**C**) CSR estimated as mean pixel intensity. Images of *C. elegans* by fluorescence microscopy (1) untreated control group, (2) pre-treated with 100 μg/mL of EGCG, (3) pre-treated with 50 μg/mL of CSE, and (4) pre-treated with 150 μg/mL of CSR; untreated control receives juglone treatment; EGCG = Epigallocatechin gallate (100 μg/mL) after juglone treatment; CST, CSE, and CSR groups receives (50, 100, and 150 μg/mL) of CST, CSE, and CSR after juglone treatment; * *p* < 0.05, ** *p* < 0.01, and *** *p* < 0.001. ns—no significance.

Heat shock proteins (HSPs) are present in all living organisms where elevated levels of HSP is highly correlated with many stress conditions comprising high temperature as well as of presence of oxidants that result in protein damage induction that in turn influences aging and longevity as well [29]. Suppression of hsp-16.2/GFP expression in *C. elegans* pretreated with the extract and different fractions of *C. speciosum* leaves was estimated using transgenic strain TJ375 that expresses hsp-16.2/GFP upon exposure to juglone treatment. Treatment of the transgenic strain TJ375 with different concentrations (50, 100, and 150 μg/mL) of extract and fractions of *C. speciosum* leaves elicited a substantial reduction in the expression of hsp-16.2/GFP as evidenced by fluorescence microscopy analyses in a dose dependent manner that is in turn correlated with the ability of the tested samples to enhance the worm survival rate and to reduce the intracellular ROS (Figure 3). Images showed that the worms treated with 150 μg/mL of CSR exerted the lower fluorescence intensity with respect to the untreated control group displaying 40.43% reduction in fluorescence with respect to the untreated group meanwhile that pretreated with 100 μg/mL of EGCG (epigallocatechin gallate) revealed 82.79% decrease in fluorescence. In contrast, CSE at 50 μg/mL displayed the lowest reduction in fluorescence with respect to all of the examined samples estimated by 7.14%. CST, CSE, and CSR exerted 15.06%, 7.14%, and 15.23% reduction in fluorescence compared to untreated group 50 μg/mL, respectively; meanwhile, they showed 16.73%, 15.35%, and 20.48% reduction in fluorescence compared to untreated group 100 μg/mL, respectively, whereas they exhibited 17.68%, 22.12%, and 40.43% reduction in fluorescence compared to untreated group 150 μg/mL, respectively.

### 3.4. Phytochemical Profiling of C. speciosum Leaves Total Methanol Extract Using LC/ESI/MS-MS

**Figure 4:**
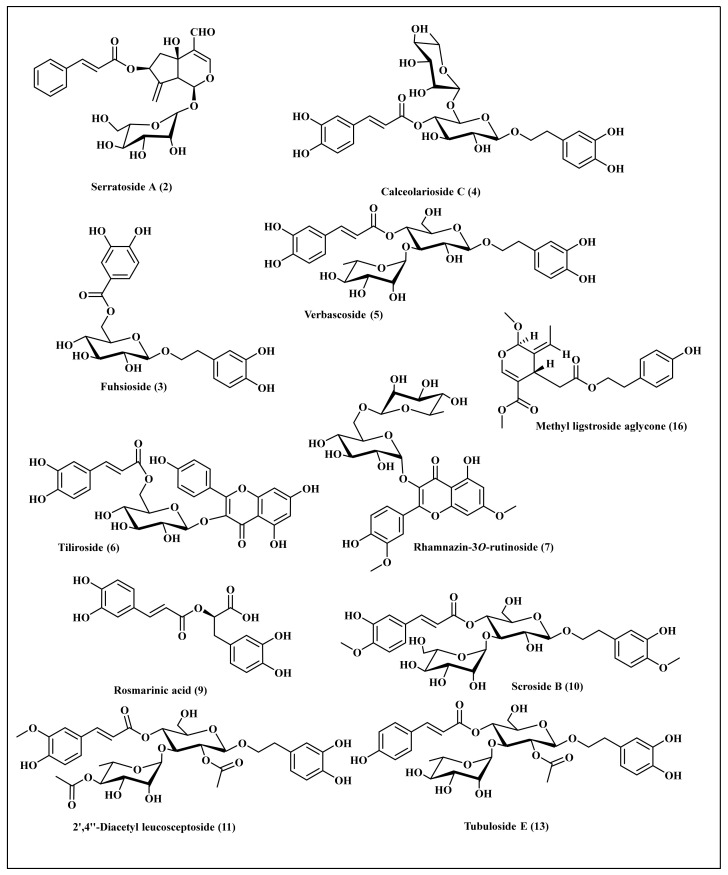
Major compounds tentatively identified in the total methanol extract of leaves from *C. speciosum* using HPLC-ESI-MS/MS in the negative ion mode.

Phytochemical investigation of *C. speciosum* leaves total methanol extract that showed the highest survival rate towards *C. elegans* with considerable antioxidant behavior as revealed from both in vitro and in vivo assays was performed tentatively using LC/ESI/MS-MS techniques. Tentative metabolite assignments were done via comparing molecular ions of [M − H]^−^ in the negative ionization mode in addition to lower *m/z* fragment ions resulting from MS/MS fragmentation with previously reported data alongside with online public databases. Besides, the sequential loss of sugar groups enabled the identification of the aglycone molecular weight with consequent identification of the various glycosides. It is worthy to highlight that this is the first comprehensive metabolites profiling performed on *C. speciosum* leaves using LC/ESI/MS-MS. It revealed the presence of sixteen secondary metabolites belonging mainly to polyphenolic components where phenyl propanoids constitutes the major detected class (Table 2). Besides, phenolic acids, flavonoids and iridoids were also present. These compounds were identified as quinic acid derivative **(1)** [30], serratoside A **(2)** [31,32], fuhsioside **(3)** [33], calceolarioside C **(4)** [34], verbascoside **(5)** [35], tiliroside **(6)** [36], rhamnazin-3*O*-rutinoside **(7)** [35], quercetin methyl galloyl-hexoside **(8)** [37], rosmarinic acid **(9)** [35], scroside B (**10**) [38], 2′,4″-diacetyl leucosceptoside **(11)** [39], acacetin 7-*O*-β-d-hexosyl-(1 → 2) [α-l-rhamnopyranosyl-(1 → 6)]-β-d-hexoside **(12)** [40], tubuloside E **(13)** [41], coumaric acid derivative **(14)** [42], 3-hydroxy-12-oleanene-28,29-dioic acid-3-*O*-α-L-pentosyl, 28-*O*-*β*-*D*-hexosyl ester **(15)** [43], and methyl ligstroside aglycones **(16)** [37]. A scheme showing most of the phytoconstituents tentatively identified in the total methanol extract of *C. speciosum* leaves using HPLC-ESI-MS/MS in the negative ion mode is illustrated in Figure 4.

Phenylpropanoid glycosides were identified constituting the highest abundance in *C. speciosum* leaves total methanol extract represented by compounds **(3–5)**, **(10–11)**, and **(13)** where they showed molecular ions [M − H]^−^ at *m/z* of 451, 609, 623, 667, 721, and 649, respectively, assigned to phenylpropanoid glycosides. More than one type of phenylpropanoid glycosides exist in the total methanol extract in which one type represented by verbascoside consists of caffeoyl and dihydroxy phenylethanol moieties linked together with two sugars, hexose and deoxy hexose. The deoxy hexose moiety of verbascoside is rhamnose. Another type represented by calceolarioside C consists of caffeoyl and dihydroxy phenylethanol moieties linked together with two sugars, hexose and pentose. In MS/MS fragmentation, both types are characterized by elimination of 162 atomic mass units (amu) corresponding to caffeoyl ion and the appearance of a fragment at *m/z* 161. Meanwhile another type of phenylpropanoid glycosides scroside B is characterized by the presence of feruloyl and hydroxy methoxy phenylethanol moities linked together with two hexose sugar moieties where its MS/MS analysis by elimination of 176 amu corresponding to feruloyl moiety and the appearance of a fragment at *m/z* 175. Furthermore an additional type of phenylpropanoid glycosides, 2′,4″-diacetyl leucosceptoside **(11)**, is characterized by the presence of caffeoyl and dihydroxy phenylethanol moieties linked together with two acetylated sugars, hexose and deoxy hexose, and it is characterized by elimination of 162 atomic mass units (amu) corresponding to caffeoyl ion and the appearance of a fragment at *m/z* 161. Meanwhile tubuloside E is characterized by the presence of coumaroyl and dihydroxy phenylethanol moieties linked together with two sugars, an acetylated hexose and deoxy hexose moieties and showed fragmentation pattern at *m/z* 163 which is characteristic to coumaric acid. However, four flavonoid glycosides were detected in total methanol extract of *C. speciosum* leaves represented by tiliroside, rhamnazin-3*O*-rutinoside, quercetin methyl galloyl-hexoside, and acacetin trioside. Rhamnazin-3-*O*-rutinoside was detected as a flavonol linked to a disaccharide, rutinose, at position 3 displaying molecular ion peak [M − H]^−^ at *m/z* of 637.

Meanwhile, quercetin methyl galloyl-hexoside and tiliroside revealed molecular ion peaks [M − H]^−^ at *m/z* of 629 and 593, respectively, showing fragment ions in MS/MS fragmentation of 301 and 285, respectively, corresponding to quercetin aglycones in the former and kaempferol in the latter. Regarding the existence of phenolic acids and their derivatives in the in *C. speciosum* leaves total methanol extract quinic acid derivative, rosmarinic acid and coumaric acid derivative were detected at molecular ions [M − H]^−^ at *m/z* of 569, 359, and 561, respectively. Rosmarinic acid is an ester composing of caffeic acid and 3, 4-dihydroxyphenyl lactic acid and it is of a wide abundance in members of Lamiaceae and Boraginaceae families. Herein, it showed [M − H]^−^ at *m/z* of 359 with MS/MS analysis showing fragments at 197, 179, 161, and 133. The richness of *C. speciosum* leaves with phenolic compounds as clarified from LC/MS analysis further confirms the antioxidant potential of the plant.

The results of LC/MS analysis of *C. speciosum* comes in accordance with other previously investigated *Clerodendrum species* where phenylpropanoids constitute the major observed class of secondary metabolites. Phenylpropanoid glycosides were isolated in considerable quantities from several Lamiaceae species as shown by Erdtman and are considered as crucial taxonomic markers in members of Lamiaceae [44]. LC/MS analysis previously conducted on *C. inerme* and *C. splendens* revealed that phenylpropanoids, flavonoids, phenolic acids, iridoid glycosides, as well as diterpenoids and fatty acid derivatives represents the major classes of secondary metabolites. Verbascoside, markhamioside B, magnoloside A or D, as well as markhamioside are the predominate phenylpropanoid in *C. inerme* and *C. splendens.* However*,* rhamnazin-3-*O*-rutinoside was identified in the leaves of *C. splendens* in addition rosmarinic acid was detected only in the methanol extract of the leaves of *C. splendens* that further confirms the relation between our studied *C. speciosum* and *C. splendens.* Besides, flavonoids such as scutellarein, naringenin, 4′-methyl scutellarein-7-*O*-hexuronide, and acacetin-7-*O*-hexuronide were identified in *C. inerme* leaves methanol extract [35]. Furthermore, phenylpropanoids represented by isoacteoside, acteoside martynoside, leucosceptoside A, incanoside C, jionoside C, and jionoside D were αpreviously identified from *C. infortunatum* in addition to the presence of acetylated phenylpropanoids such as 2″-O-acetyl-martyonside and 3″-O-acetyl-martyonside which further confirms our results. Regarding flavonoids, apigenin 7-O-glucuronide and acacetin 7-O-glucuronide were also present in *C. infortunatum* [45].

### 3.5. Computer Aided Drug Design Studies

#### 3.5.1. In Silico Molecular Docking

Molecular docking was done for all of the identified compounds from *C. speciosum* leaves total methanol extract within the active sites of DAF-2 protein where survival and combating oxidative stress is highly crucial for many organisms as this proceeds via the stimulation of various signaling pathways. DAF-2 protein is an insulin receptor-like protein that was linked to the antioxidant potentiality of *Caenorhabditis elegans* in several reports [46,47]. *Caenorhabditis elegans* depends on daf-2 protein to regulate longevity and resist oxidative stress [48]. Consequently, daf-2 protein (PDB ID: 4JDE) has been utilized as a target in our docking study using Naringenin as a reference ligand due to its reported activities [49,50]. In this study, we depended on the binding free energy (**∆G)** between the docked molecules and the active site, in addition to the correct binding mode. The binding free energies were summarized in Table 3. Results of molecular docking illustrated in Table 3 revealed that rosmarinic acid **(9)** followed by fuhsioside **(3)** showed the best fitting within the active sites of DAF-2 protein employing both pH-based and rule-based ionization modes.

**Figure 5:**
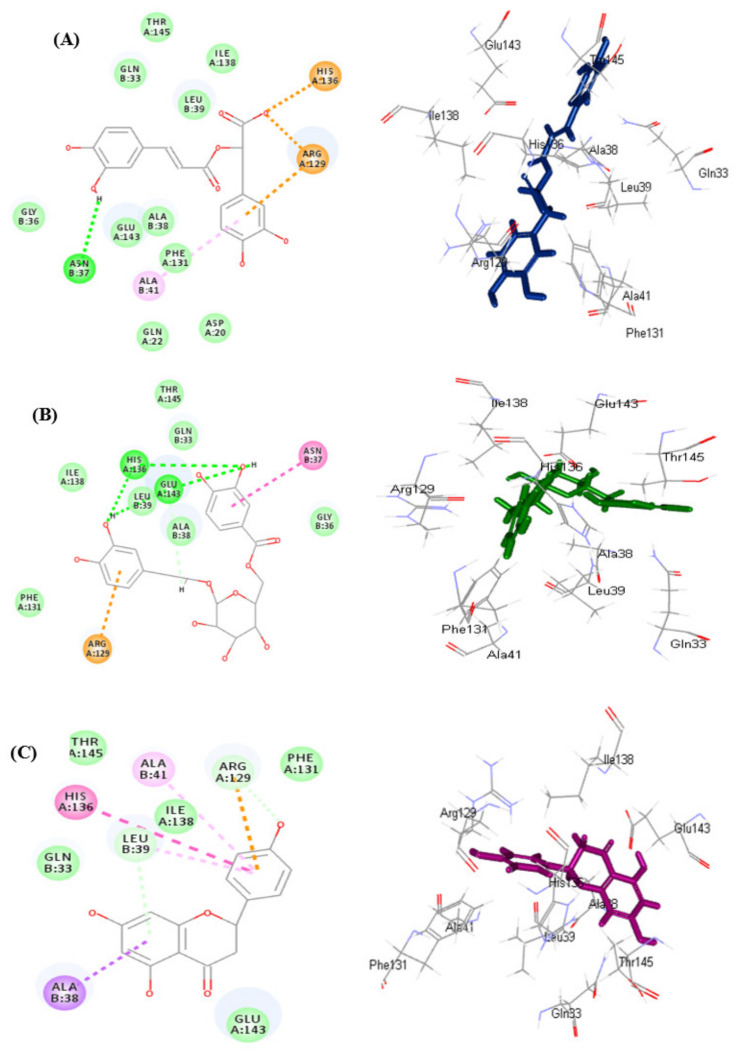
2D and 3D of rosmarinic acid (**A**), fuhsioside (**B**), and naringenin (**C**) within the active sites of daf2 protein employing pH-based ionization mode; H-bonds (heavy green dotted bonds); C-H bonds (light green dotted bonds); π-ionic bonds and attractive charges (orange dotted bonds); π-δ bond (violet dotted bonds); π-π bonds (heavy purple dotted bonds) and π-alkyl bonds (light purple dotted bonds).

They showed binding energies (∆G) of −41.99 and −17.70 kcal/mole, respectively, in pH-based ionization mode meanwhile they exerted ∆G estimated by −41.24 and −28.73 kcal/mol, respectively, in rule-based ionization mode. Employing pH-based ionization mode that actually mimic the physiological pH, the tight fitting of rosmarinic acid **(9)** to the active site, is attributed to the formation of multiple bonds manifested by one conventional H-bond with Asn37; 1 π-cation interaction with Arg129 in addition to two ionic salt bridge formation and two attractive charges between the carboxylic acid group and His136 and Arg129 amino acid residue (Figure 5A). Meanwhile fuhsioside **(3)** forms four conventional H-bonds with His136, Leu39, Glu143; one C-H bond with Ala38; one π-cation interaction with Arg129 in addition to the formation of an amide −π stacked bond (Figure 5B). The binding mode of naringenin as a refrence ligand showed a binding free energy of −19.45 kcal/mol. It forms one π-δ bond with Ala38; one π-cation bond with Arg129; two C-H bonds with Leu39 and Arg129; two π-alkyl bonds with Ala41 and Leu39 in addition to π-π-T-shaped bond with His136 (Figure 5C).

**Figure 6:**
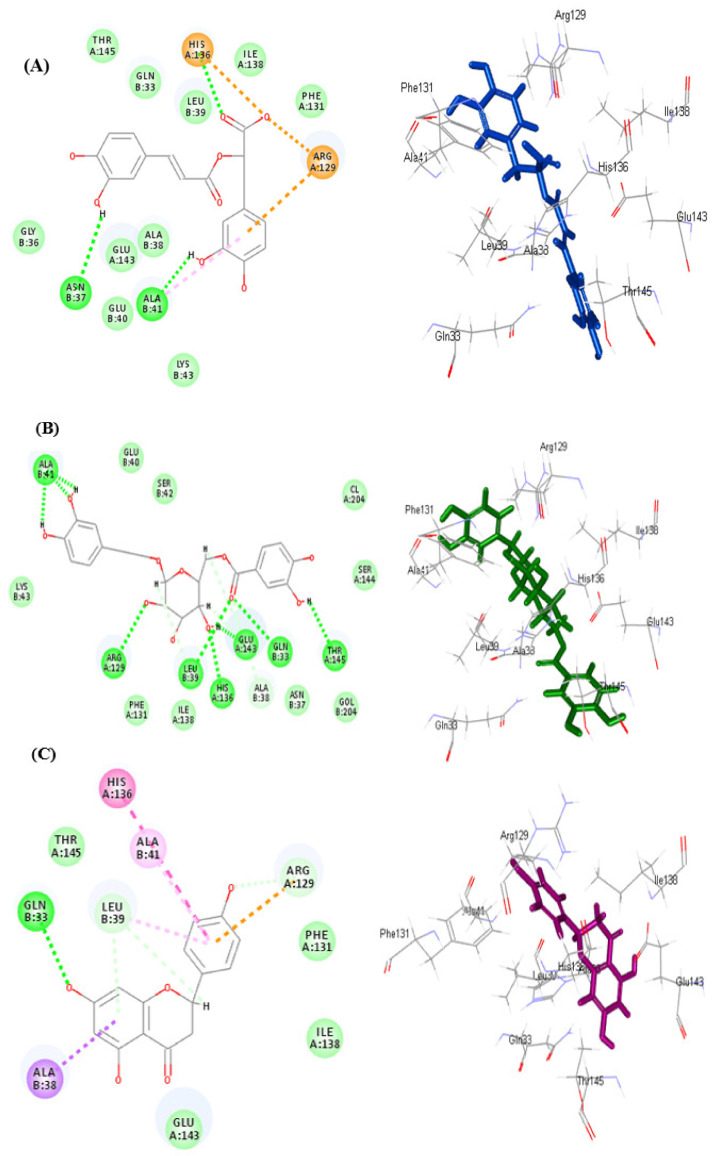
2D and 3D of rosmarinic acid (**A**), fuhsioside (**B**), and naringenin (**C**) within the active sites of daf2 protein employing rule-based ionization mode; H-bonds (heavy green dotted bonds); C-H bonds (light green dotted bonds); π-ionic bonds and attractive charges (orange dotted bonds); π-δ bond (violet dotted bonds); π-π bonds (heavy purple dotted bonds) and π-alkyl bonds (light purple dotted bonds).

Regarding rule-based ionization mode, rosmarinic acid **(9)** forms multiple bonds with the active center represented by two hydrogen bonds with Asn37, and ALa41; one π-alkyl bonds with Ala41; three π-anion, π-cation and attractive charges with Arg129 and His136 with the aromatic ring and the carboxylic acid moiety of the compound (Figure 6A). However, fuhsioside **(3)** forms nine hydrogen bonds with His136, Leu39, Glu143, Gln33, Ala41, Arg129, Thr145; two C-H bonds Glu 143, and Leu39 (Figure 6B). Regarding naringenin, it forms one conventional H-bond with Gln33; one π-δ bond with Ala38; one π-cation bond with Arg129; three C-H bonds with with Leu39 and Arg129; one π-alkyl bonds with Leu39 in addition to π-π-T-shaped bond with His136 (Figure 6C).

**Figure 7:**
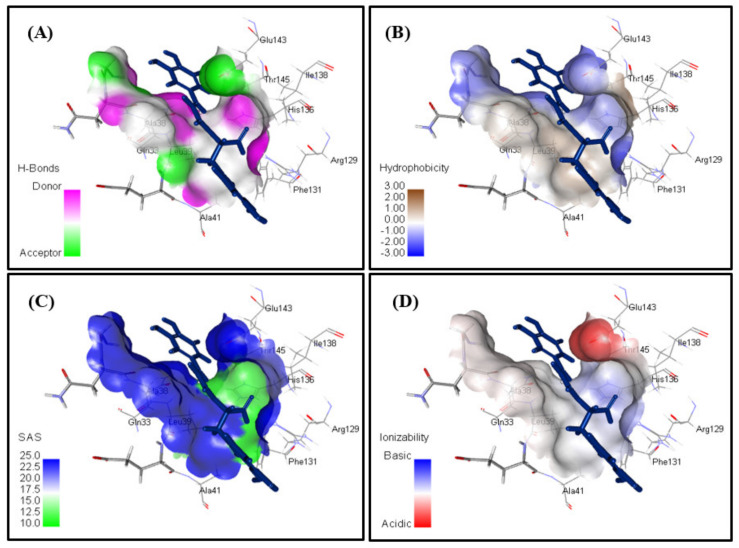
The presence of rosmarinic acid within the active pocket of Daf-2 protein illustrating (**A**) hydrogen bond type, with receptor donors in green and receptor acceptors in cyan.; (**B**) hydrophobicity of the receptor residues, from blue for hydrophilic to brown for hydrophobic; (**C**) solvent accessibility of the receptor residues from blue for exposed to green for buried; and (**D**) the ionizability of the receptor residues, from blue for basic to red for acidic.

Besides, the presence of rosmarinic acid within the active pocket of Daf-2 protein illustrating hydrogen bond type, with receptor donors in green and receptor acceptors in cyan.; hydrophobicity of the receptor residues, from blue for hydrophilic to brown for hydrophobic; solvent accessibility of the receptor residues from blue for exposed to green for buried and the ionizability of the receptor residues, from blue for basic to red for acidic is displayed in Figure 7. Rosmarinic acid was previously proved to act as a potent natural antioxidant evidenced by many assays as DPPH and ABTS assays showing 72.3 ± 3.3 μM as IC_50_ in the former and 3.7 ± 0.7 mM Trolox as TEAC in the latter. It acts as free radicle scavenger besides, it showed a significant activity versus lipid peroxidation [51,52].

#### 3.5.2. ADME/TOPAKT Prediction

**Figure 8:**
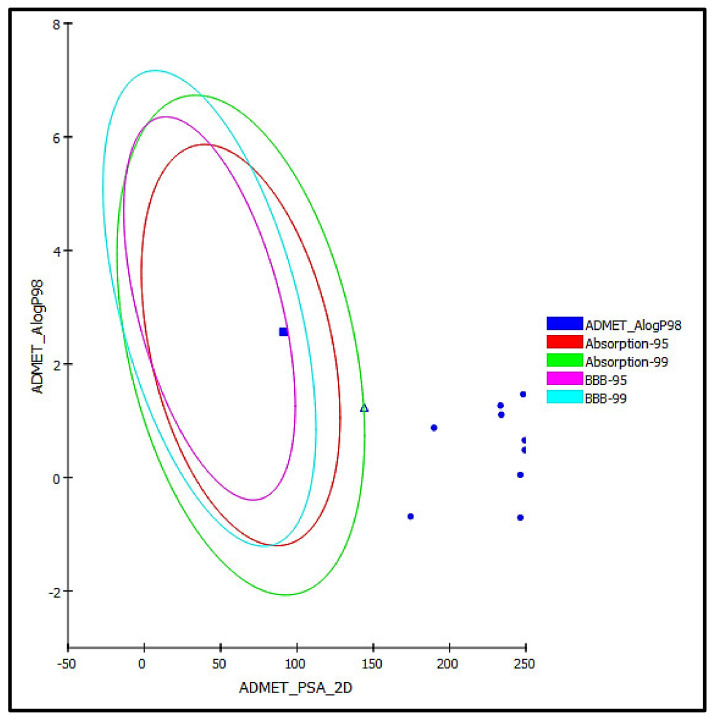
ADMET Plot for major compounds identified in the total methanol extract of *C. speciosum* showing 95% and 99% confidence limit ellipses with respect to the human intestinal absorption and the blood–brain barrier (BBB) models; rosmarinic acid **(9)** (triangle) and methyl ligstroside aglycone **(16)** (large filled square).

The identified compounds from *C. speciosum* leaves total methanol extract were subjected to ADME/TOPAKT estimation in an effort to evaluate their pharmacokinetic, pharmacodynamic, and toxicity behavior as revealed by theoretical estimates performed by Discovery Studio 4.5 (Accelrys Inc., San Diego, CA, USA) built in protocol. Results displayed in Table 4 showed that methyl ligstroside aglycone **(16)** showed good intestinal absorption, whereas rosmarinic acid **(9)** revealed low human intestinal absorption and thus lies within the 99% absorption ellipse as shown in ADMET plot (Figure 8). In contrast all other identified compounds showed very low absorption characteristics and consequently lie outside in the 99% absorption ellipse in ADMET plot. Besides, all the identified compounds showed undefined BBB behavior lying outside BBB 99% confidence eclipse, whereas methyl ligstroside aglycones showed low penetration via BBB and thus present within the 95% confidence eclipse of BBB. Concerning the solubility level, serratoside A **(2)** showed optimal solubility; meanwhile, fuhsioside **(3),** rosmarinic acid **(9),** and methyl ligstroside aglycone **(16)** exhibited good solubility, whereas other compounds showed low solubility pattern. Regarding plasma protein binding, all of the examined compounds revealed less than 90% binding. Besides, they showed no inhibition to CPY2D6 and did not exhibit hepatotoxicity (Table 4).

Regarding TOPKAT analyses, all the examined compounds showed to be non-mutagenic in Ames prediction without carcinogenic effects in both male and female rats NTP except for rhamnazin-3*O*-rutinoside **(7)** that revealed certain carcinogenicity towards male rats NTP. In addition, all of the tested compounds revealed mild to none ocular and skin irritation except tiliroside **(6)** that exerted a moderate ocular irritation. Besides, the compounds displayed rat oral LD50 values in the range of 0.67–10.57 g/kg body wt. where fuhsioside **(3)** and rosmarinic acid **(9)** showed LD50 of 5.82 and 3.17 g/kg body wt., respectively. Similarly, the analyzed compounds showed rat chronic LOAEL (The lowest-observed-adverse-effect level) in the range of 0.01–0.16 g/kg body wt. where fuhsioside **(3)** and rosmarinic acid **(9)** showed LOAEL of 0.16 and 0.13 g/kg body wt., respectively, that in turn reflects their safety (Table 5). From ADME/TOPAKT analyses, it was clearly obvious that fuhsioside **(3)** and rosmarinic acid **(9)** that showed the highest docking scores reflecting their high antioxidant potential. Besides, they displayed considerable safety profile evidenced by their toxicity criteria with limited pharmacokinetic and pharmacodynamic parameters. Meanwhile, certain treatment is required to slightly enhance their pharmacokinetic and pharmacodynamic parameters and thus can be formulated with *C. speciosum* leaves in pharmaceutical dosage forms to alleviate oxidative stress.

## 4. Conclusions

From the foregoing study, it can be concluded that a methanol extract from leaves of *C. speciosum* effectively counteract oxidative stress as confirmed by the in vitro, in vivo studies that were further ascertained by molecular docking experiment. The main active substances are polyphenols, especially phenyl propanoids as major class of secondary metabolites. Besides, phenolic acids, flavonoids and iridoids were also present. Rosmarinic acid showed the best fitting within the active sites of Daf-2 protein followed by fuhsioside exceeding that of naringenin. Besides, they displayed considerable safety profile evidenced by their toxicity criteria with limited pharmacokinetic and pharmacodynamic parameters. Meanwhile, certain treatment is required to slightly enhance their pharmacokinetic, and pharmacodynamic parameters and thus can be formulated with *C. speciosum* leaves in pharmaceutical dosage forms to alleviate oxidative stress. Noteworthy to highlight that this is the first study to be conducted on *C. specioum* leaves. In depth in vivo studies accompanied by clinical trials are necessary to further confirm the obtained results.

## Figures and Tables

**Table 1:** In vitro antioxidant determination of the extract and different fractions of *C. speciosum* leaves using (DPPH^•^) and FRAP.

Sample	DPPH (EC_50_ µg/mL)	FRAP (Fe^2+^ Equivalents/mg of Sample)
CST	27.1 ± 1.7	11.44 ± 0.68
CSE	16.2 ± 1.2	16.27 ± 0.86
CSR	21.3 ± 2.0	12.16 ± 0.68
Ascorbic acid	2.92 ± 0.29	-
Quercetin	-	24.04 ± 1.23

**Table 2:** Phytoconstituents tentatively identified in the total methanol extract of *C. speciosum* leaves using HPLC-ESI-MS/MS in the negative ion mode.

Peak No.	*t_R_* (min)	Name	[M − H]^–^ (*m/z*)	MS^2^ (*m/z*)	References
**1**	8.10	Quinic acid derivative	569	389, 371, 327, 265, 191, 173	[30]
**2**	16.10	7-Cinnamoyloxyugandoside (Serratoside A)	503	435, 341, 241, 179, 163	[31,32]
**3**	22.81	Fuhsioside	451	405, 315, 345	[33]
**4**	23.33	Calceolarioside C	609	179, 161	[34]
**5**	27.70	Verbascoside	623	605, 577, 517, 507, 461, 179, 161	[35]
**6**	31.85	Tiliroside	593	447, 285	[36]
**7**	33.09	Rhamnazin-3-*O*-rutinoside	637	475, 329, 297	[35]
**8**	36.42	Quercetin methyl galloyl-hexoside	629	179, 301, 463, 477	[37]
**9**	38.20	Rosmarinic acid	359	197, 179, 161, 133	[35]
**10**	39.66	Scroside B	667	623, 505, 491, 461, 401, 329, 193, 175	[38]
**11**	81.18	2′,4″-Diacetyl leucosceptoside	721	175, 193	[39]
**12**	83.19	Acacetin-7-*O*-β-d-hexosyl-(1 → 2)[α-l-rhamnopyranosyl-(1 → 6)]-β-d-hexoside (Acacetin trioside)	723	283	[40]
**13**	87.92	Tubuloside E	649	147, 351, 497	[41]
**14**	90.70	Coumaric acid derivative	561	439, 163	[42]
**15**	99.39	3-Hydroxy-12-oleanene-28,29-dioic acid 3-*O*-α-L-pentosyl, 28-*O*-*β*-*D*-hexosyl ester	779	733	[43]
**16**	118.54	Methyl ligstroside aglycone	375	361	[37]

**Table 3:** Binding energies (kcal/mole) of major identified phytoconstituents present in the total methanol extract of *C. speciosum* leaves within the active sites of daf-2 protein employing both pH-based and rule-based ionization modes.

Compounds	pH-Based Ionization Mode	Number of Formed Hydrogen Bonds with the Amino Acid Residues	Rule-Based Ionization Mode	Number of Formed Hydrogen Bonds with the Amino Acid Residues
Serratoside A **(2)**	39.83	-	27.20	4; Ala41, Arg129, Lys43
Fuhsioside **(3)**	−17.7	4; His136, Leu39, Glu143	−28.73	9; His136, Leu39, Glu143, Gln33, Ala41, Arg129, Thr145
Calceolarioside C **(4)**	FD	-	FD	-
Verbascoside **(5)**	FD	-	FD	-
Tiliroside **(6)**	−17.5	6; Gln33, Ala41, His136, Leu39, Glu143	FD	-
Rhamnazin-3*O*-rutinoside **(7)**	FD	-	FD	-
Rosmarinic acid **(9)**	−41.99	1; Asn37	−41.24	1; Asn37
Scroside B **(10)**	FD	-	FD	-
2,4 Diacetyl leucopstoside **(11)**	FD	-	FD	-
Tubuloside E **(13)**	FD	-	FD	-
Methyl ligstroside aglycone **(16)**	−0.19	3; His136, Leu39, Glu143	−3.34	2; Gly36, Gln33
Naringenin	−19.45	-	−19.31	1; Gln33

Positive values indicate unfavorable interaction; FD: fail to dock.

**Table 4:** ADMET (Absorption, distribution, metabolism, excretion, and toxicity) properties of the major compounds identified in the total methanol extract of *C. speciosum* leaves.

Compounds	Absorption Level	Solubility Level	BBB Level	PPB Level	CPY2D6	Hepatotoxic	PSA-2D	Alog p98
Serratoside A **(2)**	3	4	4	False	NI	NT	174.40	−0.69
Fuhsioside **(3)**	3	3	4	False	NI	NT	189.80	0.88
Calceolarioside C **(4)**	3	2	4	False	NI	NT	249.29	0.66
Verbascoside **(5)**	3	2	4	False	NI	NT	249.29	0.48
Tiliroside **(6)**	3	2	4	False	NI	NT	233.33	1.27
Rhamnazin-3*O*-rutinoside **(7)**	3	2	4	False	NI	NT	246.34	−0.71
Rosmarinic acid **(9)**	2	3	4	False	NI	NT	144.09	1.23
Scroside B **(10)**	3	2	4	False	NI	NT	246.34	0.05
2,4 Diacetyl leucopstoside **(11)**	3	2	4	False	NI	NT	248.24	1.47
Tubuloside E **(13)**	3	2	4	False	NI	NT	233.89	1.11
Methyl ligstroside aglycone **(16)**	0	3	3	False	NI	NT	91.14	2.56

0, 1, 2, and 3 indicates good, moderate, low and very low absorption, respectively; 0, 1, 2, 3, 4, and 5 indicates extremely low, very low but possible, low, good, optimal, and too soluble, respectively; 0, 1, 2, 3, and 4 denote very high, high, medium, low, and undefined, penetration via BBB respectively. PBB, plasma protein binding; false = less than 90%, true = more than 90%; NI: non-inhibitor; NT: non-toxic.

**Table 5:** TOPKAT prediction of the major compounds identified in the total methanol extract of *C. speciosum* leaves.

Compounds	Ames Prediction	Rat Oral LD50	Rat Chronic LOAEL	Skin Irritancy	Ocular Irritancy	Rat Female NTP	Rat Male NTP
Serratoside A **(2)**	Non-Mutagen	0.77	0.01	Mild	None	Non-Carcinogen	Non-Carcinogen
Fuhsioside **(3)**	Non-Mutagen	5.82	0.16	None	None	Non-Carcinogen	Non-Carcinogen
Calceolarioside C **(4)**	Non-Mutagen	10.32	0.08	Mild	Mild	Non-Carcinogen	Non-Carcinogen
Verbascoside **(5)**	Non-Mutagen	10.57	0.10	Mild	Mild	Non-Carcinogen	Non-Carcinogen
Tiliroside **(6)**	Non-Mutagen	0.67	0.03	None	Moderate	Non-Carcinogen	Non-Carcinogen
Rhamnazin-3*O*-rutinoside **(7)**	Non-Mutagen	2.36	0.04	Mild	Mild	Non-Carcinogen	Carcinogen
Rosmarinic acid **(9)**	Non-Mutagen	3.17	0.13	Mild	Mild	Non-Carcinogen	Non-Carcinogen
Scroside B **(10)**	Non-Mutagen	9.60	0.04	Mild	Mild	Non-Carcinogen	Non-Carcinogen
2,4 Diacetyl leucopstoside **(11)**	Non-Mutagen	8.46	0.05	Mild	Mild	Non-Carcinogen	Non-Carcinogen
Tubuloside E **(13)**	Non-Mutagen	8.97	0.10	Mild	Mild	Non-Carcinogen	Non-Carcinogen
Methyl ligstroside aglycone **(16)**	Non-Mutagen	2.11	0.03	Mild	Mild	Non-Carcinogen	Non-Carcinogen

Both Rat oral LD50 and Rat Chronic LOAEL are expressed in g/kg body weight.

## Data Availability

The data presented in this study are available in this manuscript.

## References

[1] Youssef F.S., Labib R.M., Eldahshan O.A. (2017). Synergistic hepatoprotective and antioxidant effect of artichoke, fig, mulberry herbal mixture on HepG2 cells and their metabolic profiling using NMR coupled with chemometrics. Chem. Biodivers..

[2] Sies H., Berndt C., Jones D.P. (2017). Oxidative stress. Annu. Rev. Biochem..

[3] Ashour M.L., Youssef F.S., Gad H.A., El-Readi M.Z., Bouzabata A., Abuzeid R.M., Sobeh M., Wink M. (2018). Evidence for the anti-inflammatory activity of *Bupleurum marginatum* (Apiaceae) extracts using in vitro and in vivo experiments supported by virtual screening. J. Pharm. Pharmacol..

[4] Reuter S., Gupta S.C., Chaturvedi M.M., Aggarwal B.B. (2010). Oxidative stress, inflammation, and cancer: How are they linked?. Free Radic. Biol. Med..

[5] Chen W., Sudji I.R., Wang E., Joubert E., van Wyk B.-E., Wink M. (2013). Ameliorative effect of aspalathin from rooibos (*Aspalathus linearis*) on acute oxidative stress in *Caenorhabditis elegans*. Phytomedicine.

[6] Chen W., Rezaizadehnajafi L., Wink M. (2013). Influence of resveratrol on oxidative stress resistance and life span in Caenorhabditis elegans. J. Pharm. Pharmacol..

[7] Ayuda-Durán B., González-Manzano S., González-Paramás A.M., Santos-Buelga C. (2020). *Caenorhabditis elegans* as a model organism to evaluate the antioxidant effects of phytochemicals. Molecules.

[8] Abbas S., Wink M. (2009). Epigallocatechin gallate from green tea (*Camellia sinensis*) increases lifespan and stress resistance in *Caenorhabditis elegans*. Planta Med..

[9] Abbas S., Wink M. (2010). Epigallocatechin gallate inhibits beta amyloid oligomerization in *Caenorhabditis elegans* and affects the daf-2/insulin-like signaling pathway. Phytomedicine.

[10] Kar P., Goyal A., Das A., Sen A. (2014). Antioxidant and pharmaceutical potential of *Clerodendrum* L.: An overview. Int. J. Green Pharm..

[11] Okwu D.E., Iroabuchi F. (2009). Phytochemical composition and biological activities of *Uvaria chamae* and *Clerodendoron splendens*. J. Chem..

[12] Bum E.N., Taiwe G., Moto F., Ngoupaye G., Vougat R., Sakoue V., Gwa C., Ayissi E., Dong C., Rakotonirina A. (2011). Antiepileptic medicinal plants used in traditional medicine to treat epilepsy. Clinical and Genetic Aspects of Epilepsy.

[13] Zhang Q., Zhang J., Shen J., Silva A., Dennis D.A., Barrow C.J. (2006). A simple 96-well microplate method for estimation of total polyphenol content in seaweeds. J. Appl. Phycol..

[14] Blois M.S. (1958). Antioxidant determinations by the use of a stable free radical. Nature.

[15] Benzie I.F., Strain J.J. (1996). The ferric reducing ability of plasma (FRAP) as a measure of “antioxidant power”: The FRAP assay. Anal. Biochem..

[16] Stiernagle T. (2021). Maintenance of *Caenorhabditis elegans*. Int. J. Biomed. Health Sci..

[17] Abbas S., Wink M. (2014). Green tea extract induces the resistance of *Caenorhabditis elegans* against oxidative stress. Antioxidants.

[18] Sobeh M., ElHawary E., Peixoto H., Labib R.M., Handoussa H., Swilam N., El-Khatib A.H., Sharapov F., Mohamed T., Krstin S. (2016). Identification of phenolic secondary metabolites from *Schotia brachypetala* Sond.(Fabaceae) and demonstration of their antioxidant activities in *Caenorhabditis elegans*. PeerJ.

[19] Sobeh M., Youssef F.S., Esmat A., Petruk G., El-Khatib A.H., Monti D.M., Ashour M.L., Wink M. (2018). High resolution UPLC-MS/MS profiling of polyphenolics in the methanol extract of *Syzygium samarangense* leaves and its hepatoprotective activity in rats with CCl_4_-induced hepatic damage. Food Chem. Toxicol..

[20] Janibekov A.A., Youssef F.S., Ashour M.L., Mamadalieva N.Z. (2018). New flavonoid glycosides from two *Astragalus* species (Fabaceae) and validation of their antihyperglycaemic activity using molecular modelling and in vitro studies. Ind. Crops Prod..

[21] Youssef F.S., Ovidi E., Musayeib N.M.A., Ashour M.L. (2021). Morphology, anatomy and secondary metabolites investigations of *Premna odorata* Blanco and evaluation of its anti-tuberculosis activity using in vitro and in silico studies. Plants.

[22] Mamadalieva N.Z., Youssef F.S., Hussain H., Zengin G., Mollica A., Al Musayeib N.M., Ashour M.L., Westermann B., Wessjohann L.A. (2021). Validation of the antioxidant and enzyme inhibitory potential of selected triterpenes using in vitro and in silico studies, and the evaluation of their ADMET properties. Molecules.

[23] Baba S.A., Malik S.A. (2015). Determination of total phenolic and flavonoid content, antimicrobial and antioxidant activity of a root extract of Arisaema jacquemontii Blume. J. Taibah Univ. Sci..

[24] Wink M. (2015). Modes of action of herbal medicines and plant secondary metabolites. Medicines.

[25] Van Wyk B.-E., Wink M. (2017). Medicinal Plants of the World.

[26] Prior R.L., Wu X., Schaich K. (2005). Standardized methods for the determination of antioxidant capacity and phenolics in foods and dietary supplements. J. Agric. Food Chem..

[27] Aboulwafa M.M., Youssef F.S., Gad H.A., Sarker S.D., Nahar L., Al-Azizi M.M., Ashour M.L. (2018). Authentication and discrimination of green tea samples using UV-Visible, FTIR and HPLC techniques coupled with chemometrics analysis. J. Pharm. Biomed. Anal..

[28] Saling S.C., Comar J.F., Mito M.S., Peralta R.M., Bracht A. (2011). Actions of juglone on energy metabolism in the rat liver. Toxicol. Appl. Pharmacol..

[29] Swindell W.R. (2009). Heat shock proteins in long-lived worms and mice with insulin/insulin-like signaling mutations. Aging.

[30] Fernández-Poyatos M.d.P., Ruiz-Medina A., Zengin G., Llorent-Martínez E.J. (2019). Phenolic characterization, antioxidant activity, and enzyme inhibitory properties of Berberis thunbergii DC. leaves: A valuable source of phenolic acids. Molecules.

[31] Asraoui F., Kounnoun A., Cadi H.E., Cacciola F., Majdoub Y.O.E., Alibrando F., Mandolfino F., Dugo P., Mondello L., Louajri A. (2021). Phytochemical Investigation and Antioxidant Activity of *Globularia alypum* L.. Molecules.

[32] Wang J.-H., Luan F., He X.-D., Wang Y., Li M.-X. (2018). Traditional uses and pharmacological properties of Clerodendrum phytochemicals. J. Tradit. Complement. Med..

[33] Ozipek M., Saracoglu I., Kojima K., Ogihara Y., Calis I. (1999). Fuhsioside, a new phenylethanoid glucoside from Veronica fuhsii. Chem. Pharm. Bull..

[34] Nicoletti M., Galeffi C., Messana I., Marini-Bettolo G., Garbarino J., Gambaro V. (1988). Phenylpropanoid glycosides from *Calceolaria hypericina*. Phytochemistry.

[35] Elaskary H.I., Sabry O.M., Khalil A.M., El Zalabani S.M. (2020). UPLC-PDA-ESI-MS/MS Profiling of *Clerodendrum inerme* and *Clerodendrum splendens* and significant activity against *Mycobacterium tuberculosis*. Pharmacogn. J..

[36] Czerwińska M.E., Kalinowska E., Popowski D., Bazylko A. (2020). Lamalbid, chlorogenic acid, and verbascoside as tools for standardization of *Lamium album* flowers—development and validation of HPLC–DAD method. Molecules.

[37] Sobeh M., Mahmoud M.F., Petruk G., Rezq S., Ashour M.L., Youssef F.S., El-Shazly A.M., Monti D.M., Abdel-Naim A.B., Wink M. (2018). *Syzygium aqueum*: A polyphenol-rich leaf extract exhibits antioxidant, hepatoprotective, pain-killing and anti-inflammatory activities in animal models. Front. Pharmacol..

[38] Xu J., Tong C., Fu Q., Guo K., Shi S., Xiao Y. (2019). Comprehensive polyphenolic profile of *Plantago depressa* using high-speed countercurrent chromatography off-line with high-performance liquid chromatography–Diode Array Detector–Quadrupole Time-of-flight Tandem Mass Spectrometry. eFood.

[39] Munkombwe N.M. (2003). Acetylated phenolic glycosides from *Harpagophytum procumbens*. Phytochemistry.

[40] Veitch N.C., Elliott P.C., Kite G.C., Lewis G.P. (2010). Flavonoid glycosides of the black locust tree, Robinia pseudoacacia (Leguminosae). Phytochemistry.

[41] Ahn J., Chae H.-S., Chin Y.-W., Kim J. (2017). Dereplication-guided isolation of new phenylpropanoid-substituted diglycosides from *Cistanche salsa* and their inhibitory activity on NO production in macrophage. Molecules.

[42] Llorent-Martínez E.J., Spínola V., Gouveia S., Castilho P.C. (2015). HPLC-ESI-MSn characterization of phenolic compounds, terpenoid saponins, and other minor compounds in *Bituminaria bituminosa*. Ind. Crops Prod..

[43] Kasai R., Oinaka T., Yang C.-R., Zhou J., Tanaka O. (1987). Saponins from Chinese folk medicine,“Liang Wang Cha,” leaves and stems of *Nothopanax delavayi*, Araliaceae. Chem. Pharm. Bull..

[44] Cantino P.D., Sanders R.W. (1986). Subfamilial classification of Labiatae. Syst. Bot..

[45] Uddin M.J., Çiçek S.S., Willer J., Shulha O., Abdalla M.A., Sönnichsen F., Girreser U., Zidorn C. (2020). Phenylpropanoid and flavonoid glycosides from the leaves of Clerodendrum infortunatum (Lamiaceae). Biochem. Syst. Ecol..

[46] Song S., Zhang X., Wu H., Han Y., Zhang J., Ma E., Guo Y. (2014). Molecular basis for antioxidant enzymes in mediating copper detoxification in the nematode *Caenorhabditis elegans*. PLoS ONE.

[47] Barsyte D., Lovejoy D.A., LITHGOW G.J. (2001). Longevity and heavy metal resistance in daf-2 and age-1 long-lived mutants of Caenorhabditis elegans. FASEB J..

[48] Kimura K.D., Tissenbaum H.A., Liu Y., Ruvkun G. (1997). daf-2, an insulin receptor-like gene that regulates longevity and diapause in *Caenorhabditis elegans*. Science.

[49] Ge Y., Chen H., Wang J., Liu G., Cui S.W., Kang J., Jiang Y., Wang H. (2021). Naringenin prolongs lifespan and delays aging mediated by IIS and MAPK in *Caenorhabditis elegans*. Food Funct..

[50] Zhu Q., Qu Y., Zhou X.-G., Chen J.-N., Luo H.-R., Wu G.-S. (2020). A dihydroflavonoid naringin extends the lifespan of *C. elegans* and delays the progression of aging-related diseases in PD/AD models via DAF-16. Oxidative Med. Cell. Longev..

[51] Fadel O., El Kirat K., Morandat S. (2011). The natural antioxidant rosmarinic acid spontaneously penetrates membranes to inhibit lipid peroxidation in situ. Biochim. Biophys. Acta BBA-Biomembr..

[52] Cao H., Cheng W.-X., Li C., Pan X.-L., Xie X.-G., Li T.-H. (2005). DFT study on the antioxidant activity of rosmarinic acid. J. Mol. Struct..

